# Levothyroxine Replacement Improves Oxidative Status in Primary Hypothyroidism

**DOI:** 10.3389/fendo.2018.00655

**Published:** 2018-11-08

**Authors:** Laís Farias Masullo, Rejane Araújo Magalhães, Romélia Pinheiro Gonçalves Lemes, Tarcísio Paulo de Almeida Filho, Marilena Facundo de Castro, Pedro Aurio Maia Filho, Tainá Osterno Vasconcelos Cunha, Ana Rosa Pinto Quidute, Eveline Gadelha Pereira Fontenele, Guang Sun, Manoel Ricardo Alves Martins

**Affiliations:** ^1^Research Laboratory in Haemoglobinopathies and Genetics of Haematological Diseases, Federal University of Ceará, Fortaleza, Brazil; ^2^Post-Graduate Program in Pathology, Federal University of Ceará, Fortaleza, Brazil; ^3^Division of Endocrinology and Diabetes, Hospital Universitário Walter Cantídio, Federal University of Ceará, Fortaleza, Brazil; ^4^Department of Physiology and Pharmacology, Federal University of Ceará, Fortaleza, Brazil; ^5^Drug Research and Development Center-NPDM/Fortaleza, Fortaleza, Brazil; ^6^Department of Clinical Medicine, Federal University of Ceará, Fortaleza, Brazil; ^7^Discipline of Medicine, Faculty of Medicine, Memorial University, St John's, NL, Canada

**Keywords:** oxidative stress, reactive oxygen species, antioxidants, oxidants, thyroid hormones, hypothyroidism, levothyroxine replacement, thyroid disease

## Abstract

**Objective:** Although hypothyroidism has been linked to oxidative stress, data regarding the relationship between thyroid hormone levels and oxidative stress is still inconsistent. This study was designed to evaluate the effect of levothyroxine replacement on oxidative stress in women with primary hypothyroidism.

**Design:** A total of 25 female patients with primary hypothyroidism were included. Oxidative stress markers were measured before and after levothyroxine replacement treatment in all patients.

**Methods:** Oxidative stress was evaluated through the measurement of oxidants (thiobarbituric acid reactive substances [TBARS] and nitrite/nitrate levels), and antioxidants (superoxide dismutase and catalase activity).

**Results:** Antioxidant catalase activity (63.77 ± 23.8 vs. 50.12 ±12.75 atv/min; *p* = 0.03) was significantly increased and the levels of TBARS (3.02 ± 0.86 vs. 3.55 ± 0.87 μM; *p* = 0.03) were significantly decreased in the state of euthyroidism after levothyroxine replacement compared to the hypothyroidism before levothyroxine treatment. No significant change in neither nitrite/nitrate concentration (*p* = 0.18) nor in superoxide dismutase activity (*p* = 0.93) after L-T4 adjustment was found.

**Conclusions:** Our data demonstrate that levothyroxine replacement improved oxidative status in patients with primary hypothyroidism, indexed by the significantly decreased levels of malonaldehyde (MDA) and increased catalase (CAT) activity.

## Introduction

Primary hypothyroidism is a disorder defined by an increased thyroid stimulating hormone (TSH) level, which may or may not be accompanied by a decreased thyroid hormone (TH) level, T3 and T4 (1). The most common causes of hypothyroidism are autoimmune destruction of the thyroid, thyroid surgery, and radioiodine therapy (2).

Thyroid hormones play important physiological roles in differentiation, growth and metabolism, and regulate both the basal and adaptive metabolic rate (1, 3). As a result of their effects, thyroid hormones may also lead to the production of ROS and alter antioxidant mechanisms. These processes may simultaneously affect different points of oxidative stress (OS), thus creating innumerable scenarios in which the outcome is difficult to predict (4).

Hypothyroidism has been linked to cardiac dysfunctions, atherosclerosis, hypertension and coagulopathies(5, 6), which may be associated to a greater or lesser extent with oxidative stress (7–10).

A relatively small number of studies reported oxidative stress (OS) in hypothyroidism and the results are generally inconsistent. Most studies have shown increased oxidant levels (11–15), while antioxidant levels have been reported to be either increased (12, 14) or decreased (15, 16). Since most studies have compared hypothyroid patients with controls, the presence of comorbidities and interpersonal variability among patients could be a possible cause for the discrepancy in these results.

To avoid and clarify this confounding factor, we evaluated the effect of levothyroxine replacement on oxidative status in a group of female patients with primary hypothyroidism before and after the treatment. In order to minimize the interference of confounding clinical factors, all patients were evaluated in a state of hypothyroidism, then treated with levothyroxine and re-evaluated after euthyroidism was achieved.

## Participants and methods

### Study population

This study was conducted at the Endocrinology Outpatient clinic of a tertiary centre in Brazil between October 2015 and December 2016.

Our study included 25 women, aged from 23 to 59 years, diagnosed with primary hypothyroidism. Exclusion criteria consist of recent acute viral infection (less than 3 months), malignancy, liver or autoimmune disease (except Hashimoto thyroiditis), current use of antioxidant agents, vitamin supplements, tobacco use, alcohol abuse, lactation and pregnancy, and loss to follow up.

### Study design

All patients were diagnosed of hypothyroidism. They received treatment with levothyroxine and were re-evaluated to have achieved to the state of euthyroidism, after an interval of at least 6 weeks.

The treatment consisted by oral administration of synthetic levothyroxine (L-T4) 30 min before the first meal of the day. The L-T4 dose ranged from 0.8 to 2.0 μg/ kg body weight (mean: 105.74 ± 28.98 μg, from 50 to 175 μg), after an interval of at least 6 weeks (mean: 11.96 ± 8.98 weeks, from 6 to 35 weeks).

The study was conducted in accordance with the Declaration of Helsinki 2013 Brazilian version and was approved by the Ethics Research Committee of Federal University of Ceara (Comitê de ética e pesquisa da Universidade Federal do Ceará, CEP/UFC/PROPESQ, research registration number / número do parecer: 1.320.557). All participants provided written informed consent prior to participation in the study.

### Clinical and laboratory evaluation

The clinical parameters evaluated at diagnosis were body mass index (BMI), blood pressure, abdominal circumference, and heart rate. Metabolic syndrome was defined using International Diabetes Federation criteria (17).

Venous blood samples were collected from all participants in the state of both hypothyroidism and euthyroidism, after 12 h of overnight fasting. The blood samples were immediately centrifuged for 10 min at 3,500 rpm, and serum and plasma samples were isolated and immediately stored at freezer until assay. During assay, all oxidative stress parameters were measured in each sample at the same time.

The hormonal parameters analyzed were total triiodothyronine (TT3), free thyroxine (FT4), and thyrotropin (TSH). Serum TSH, FT4, and TT3 were measured with an autoanalyzer Advia Centaur®, using the electrochemiluminescence immunoassay method. Euthyroidism was defined as TSH between 0.34 and 4.5 mUI/L, FT4 between 0.61, and 1.48 ng/dL and TT3 between 0.7 and 2.04 ng/dL. Hypothyroidism was defined as a serum TSH higher than 4.5 mUI/mL (upper limit of reference range for the assay). The degree of hypothyroidism was defined as: mild hypothyroidism (TSH < 10 mIU/L) and severe hypothyroidism (TSH > 10 mIU/L) (18).

The biochemical parameters analyzed were fasting glucose, total cholesterol, and fractions, and triglycerides. Total cholesterol and triglyceride levels were measured using the enzymatic colorimetric method. High-density lipoprotein (HDL) cholesterol was measured based on the precipitation reaction of low-density lipoprotein (LDL) cholesterol and very low-density lipoprotein (VLDL), and the supernatant with HDL was measured using the enzymatic colorimetric method. LDL cholesterol was calculated with the Friedewald formula, and VLDL was calculated with triglycerides (TG)/5.

Catalase (CAT) activity was determined in haemolysate prepared through a decomposition of hydrogen peroxide at 240 nm, as described by Aebi (19). The results were expressed in atv/min. Superoxide dismutase (SOD) activity was determined in haemolysate prepared from blood collected in heparin Vacutainer tubes using a Randox kit® SD125. The results were expressed in U/mL. The NO level was determined through nitrite/nitrate levels in plasma with the colorimetric method, using a Roche kit® 11746081001, according to Green (20). The results were expressed in mg/L. The malondialdehyde (MDA) level was measured in haemolysate samples prepared using the TBARS reaction colorimetric method, as described by Draper and Hadley (21). The results were expressed in μM.

### Statistical analysis

Results are shown as mean ± s.d. To test differences between the two measurements we used the paired *t*-test or the Wilcoxon test, as appropriate. Data were analyzed using SPSS 21.0. Statistical significance was set at *p* < 0.05.

## Results

Twenty-five women (age: 42 ± 8.46 years) were included in the study. Table 1 summarizes the clinical characteristics at the start of the study.

**Table 1:** Clinical characteristics of patients included in the study.

**Variables**	**Mean ± SD**	**Range**
Age (year)	42.0 ± 8.5	23.0–56.0
AC (cm)	90.4 ± 9.2	70.0–105.0
BMI (Kg/m2)	27.2 ± 4.2	19.8–35.7
SBP (mmHg)	122.3 ± 19.2	90.0–170.0
DBP (mmHg)	79.0 ± 12.2	50.0–110.0
HR (bpm)	75.3 ± 8.4	62.0–96.0

*Age, Patient age at diagnosis; AC, Abdominal circumference; BMI, Body Mass Index; SBP, Systolic Blood Pressure; DBP, Diastolic Blood Pressure; HR, Heart Rate*.

Regarding the diagnosis of hypothyroidism, 72% (18/25) of cases was due to Hashimoto's thyroiditis, and 28% (7/25) occurred post-procedure (radioactive iodine or surgery). Concerning the degree of hypothyroidism (18), 32% (8/25) had mild hypothyroidism (TSH < 10 mIU/L) and 68% (17/25) had severe hypothyroidism (TSH> 10 mIU/L).

Regarding associated comorbidities, 48% presented with metabolic syndrome, 28% with obesity and 12.5% with hypertension at diagnosis.

As to lipid parameters improved after treatment, total cholesterol (*p* < 0.0001), LDL (*p* < 0.0001), VLDL (*p* = 0.005), and triglycerides (*p* = 0.006) were significantly lower in euthyroidism, after levothyroxine replacement. Fasting glucose and HDL were similar in both measurements (Table 2).

**Table 2:** Hormonal and biochemical measurements in hypo- and euthyroidism.

	**HYPO**	**EU**	***p-*value**
TSH (mUI/L)	31.75 ± 30.05	1.80 ± 1.18	<0.001^*^
FT4 (ng/dL)	0.65 ± 0.28	1.07 ± 0.26	<0.001^*^
T3 (ng/dL)	0.81 ± 0.33	1.01 ± 0.19	0.005^*^
GLI (mg/dL)	97.2 ± 19.90	97.72 ± 15.40	0.809
TC (mg/dL)	219.6 ± 44.30	181.92 ± 37.80	<0.0001^*^
HDL (mg/dL)	55.84 ± 12.65	52.84 ± 11.20	0.054
LDL (mg/dL)	131.26 ± 41.30	102.2 ± 28.00	<0.0001^*^
VLDL (mg/dL)	34.6 ± 16.98	28.03 ± 15.02	0.005^*^
TG (mg/dL)	187.41 ± 108.90	144.5 ± 76.45	0.006^*^

*Mean ± standard deviation*.

* *Statistical significance in comparation between hypo-and euthyroidism. HYPO, hypothyroidism; EU, euthyroidism; TSH, thyrotropin; FT4, free thyroxine; T3, total triiodothyronine; GLI, glucose; TC, total cholesterol; HDL, high-density lipoprotein; LDL, low-density lipoprotein; VLDL, very low-density lipoprotein; TG, triglycerides*.

*Reference values: TSH = 0.34–5.6; T4L = 0.61–1.48; T3 = 0.7-2.04; GLI = 60–99mg/dL, CT = desirable < 200 mg/dL, borderline = 200–240 mg/dL; HDL > 60 mg/dL; LDL = desirable < 130 mg/dL, borderline = 130–159 mg/dL; VLDL < 30 mg/dL; TG = desirable = < 130 mg/dL, borderline = 150–200 mg/dL*.

OS parameters were significantly higher in hypothyroidism, with significantly lower levels of antioxidant activity (CAT; *p* = 0.03) and significantly higher levels of oxidants (TBARS; *p* = 0.03). No significant difference was found for SOD activity and NO levels after L-T4 replacement (Table 3). These results remained statistically significant when only patients with TSH < 10 mUI/L were included in the analysis, and neither the presence of co-morbidities, including metabolic syndrome, nor the etiology of hypothyroidism, including only Hashimoto's thyroiditis, affected these findings (data not shown).

**Table 3:** Oxidative stress (OS) parameters in hypo- and euthyroidism.

	**HYPO**	**EU**	***p-*value**
CAT (atv/min)	50.25 ± 12.75	63.77 ± 23.80	0.03^*^
TBARS (10 μM)	3.55 ± 0.87	3.02 ± 0.86	0.03^*^
SOD (U/mL)	1305.5 ± 285.20	1297 ± 438.00	0.93
NO (mg/L)	36.57 ± 17.53	34.07 ± 14.90	0.18

*Mean ± standard deviation*.

* *Statistical significance in comparation between hypo-and euthyroidism. CAT, catalase; EU, euthyroidism; HYPO, hypothyroidism; NO, nitric oxide; SOD, superoxide dismutase; TBARS, thiobarbituric acid*.

## Discussion

In the present clinical study, our primary finding is that levothyroxine replacement could improve oxidative status in patients with primary hypothyroidism, indexed by significantly decreased levels of MDA and increased CAT activity after the treatment. To the best of our knowledge, this is the first study to evaluate oxidative stress in a group of female patients with primary hypothyroidism in a prospective cohort.

Previous studies regarding the influence of thyroid hormone on oxidative stress, of both transversal (13, 14, 16, 22) and prospective (11, 12, 15), have shown inconsistent results. The inconsistency may be partly due to the influence of many confounding factors known to influence OS, including individual variability. A prospective study, which is of a self-controlled design, minimizes the individual variability. Additionally, that women only in this study could potentially increase the power to detect signal because oxidative stress varies according to sex, with higher levels in women. The gender difference could be due to hormonal factors, mainly androgen activity (23–25).

The etiology of hypothyroidism may influence oxidative stress, however the majority of patients in the present study were of autoimmune origin, e.g., with same etiology in general for most participants. Our finding is supported by a recent *in vitro* study in which antioxidants treatment on blood mononuclear cells (PBMC) from patients with Hashimoto's thyroiditis, caused beneficial effects (26).

In the present study, we evaluated a number of oxidative stress markers through measuring both oxidants (TBARS and Nitrite/nitrate) and antioxidants (CAT and SOD). Oxidative stress occurs when there is an imbalance between pro-oxidants and antioxidants, which occurs when oxidants cannot be neutralized through antioxidant defenses. Oxidants are mainly produced through the mitochondrial respiratory chain, with ${\text{O}}_{2}^{-}$ produced initially in the chain. Since ${\text{O}}_{2}^{-}$ is unstable, the molecule is quickly converted to H_2_O_2_, and this process occurs through the activity of SOD, an antioxidant enzyme.

In hypothyroidism, SOD activity has not been well elucidated. Some studies reported lower activity in hypothyroidism (11, 16), while others demonstrated no significant difference of SOD activity between hypothyroidism and euthyroidism (12, 14). These differences are likely to result from variation in study design, populations, hypothyroidism severity, and associated comorbidities. Our present study found no significant difference of SOD activity levels between hypothyroidism and euthyroidism after LT-4 replacement, suggesting no significant interference from SOD' activity in antioxidant defense.

After SOD converts ${\text{O}}_{2}^{-}$ into H_2_O_2_, H_2_O_2_ can react with several cell structures, causing cell damage (27). Although H_2_O_2_ does not have an unpaired electron in the last layer, this compound is considered as a reactive oxygen species, since it can diffuse through the membrane, reacting with cellular structures and causing damage. The mechanism of neutralization occurs through CAT, which reacts with H_2_O_2_ turning it into O_2_ and H_2_O (28).

The reported results regarding CAT levels in hypothyroidism compared with controls are contradictory as well. Some reported higher (14) but some lower (16) CAT activity in patients with hypothyroidism. In our prospective study, we have demonstrated a significantly lower CAT activity in hypothyroidism which was improved after achieving euthyroidism by levothyroxine replacement. This finding suggests that the clinical condition of hypothyroidism saturates CAT activity and reduces antioxidant defense. When CAT activity is reduced in hypothyroidism, a possible excessive H_2_O_2_ in an organism could react with NO, producing peroxynitrite radicals or other hydroxyl radicals. These radicals will in turn react with cellular structures to cause damage, in a process known as lipid peroxidation (29).

MDA level was measured by TBARS reaction. TBARS is a sensitive marker of lipid peroxidation, once it is a specific degradation product. Some studies have demonstrated higher TBARS levels in hypothyroidism (11–14), whereas another study did not find significant difference in hypothyroidism (22).

Another important finding of the present study is the higher TBARS levels in hypothyroidism as compared to the euthyroid state. Lipid peroxidation in hypothyroidism may increase cardiovascular risk, since hypothyroidism is already associated with comorbidities, such as atherosclerosis and dyslipidaemia, which can be increased with oxidized LDL and OS (30). Other important findings were the beneficial effect of levothyroxine treatment on reducing lipid peroxidation and on TC, LDL, and TG levels (Table 2), known risk factors of atherosclerosis.

In the condition of inflammation, nitrite/nitrate levels are increased, which are indirect NO markers. NO is a product of an L-arginine and oxide nitric synthase (NOS) reaction, occurring in different cells. Although NO does not display marked toxicity, it reacts rapidly with the superoxide radical, the initiator of the oxidative cascade, therefore generating highly reactive and damaging peroxynitrite radicals (31, 32).

Some studies have shown increased levels of NO in hypothyroidism when compared with controls (11, 22). However, when compared in a prospective study, no significant difference was found (11), which is consistent with the finding from the present study.

Due to our strict exclusion criteria, we were able to include only a small sample of patients. However, this strict study design enabled us to have a more homogeneous group of patients, and hence, greatly reduced the influence from major confounding factors. In addition, the prospective design of the study also increased the reliability of the findings.

In conclusion, our data demonstrate beneficial effects of levothyroxine replacement on the oxidative status and lipid profile in hypothyroid women. The beneficial effects should be considered when evaluating early TH replacement in primary hypothyroidism in order to minimize complications associated with the lipid profile and OS in the long term. These findings open the way to new studies associating complications commonly observed in patients with hypothyroidism and oxidative stress.

## Author contributions

LM and RM: data collection and scientific supervision; MM and RL: study design and scientific supervision; LM, TdA, MdC, and PM: laboratory analysis; LM, MM, TdA: writing manuscript; TC, EF, AQ, and GS: manuscript review; LM, MM: organization of the database and statistical treatment.

### Conflict of interest statement

The authors declare that the research was conducted in the absence of any commercial or financial relationships that could be construed as a potential conflict of interest.

## Abbreviations

BMI: Body mass index
CAT: Catalase
FT4: Free thyroxine
HDL: High-density lipoprotein
LDL: Low-density lipoprotein
LT-4: Levothyroxine
MDA: Malondialdehyde
NO: Nitric oxide
OS: Oxidative stress
${\text{O}}_{2}^{-}$: Superoxide anion
ROS: Reactive oxygen species
SOD: Superoxide dismutase
TSH: Thyrotropin
TT3: Total triiodothyronine
TG: Triglycerides
VLDL: Very low-density lipoprotein.
